# Association between frailty and breast cancer incidence and construction of a breast cancer prediction model based on machine learning

**DOI:** 10.3389/fpubh.2026.1858164

**Published:** 2026-07-01

**Authors:** Nani Li, Jian Liu, Minjing You, Yiran Chen, Mulan Chen, Weiwei Huang, Jing Huang, Fan Wu

**Affiliations:** 1 Department of Breast Medical Oncology, Clinical Oncology School of Fujian Medical University, Fujian Cancer Hospital, Fuzhou, Fujian, China; 2 Department of Radiation Oncology, Clinical Oncology School of Fujian Medical University, Fujian Cancer Hospital, Fuzhou, Fujian, China; 3 Department of Pharmacy, Clinical Oncology School of Fujian Medical University, Fujian Cancer Hospital, Fuzhou, Fujian, China

**Keywords:** breast cancer, frailty, frailty index, machine learning, NHANES, prediction model, SHAP analysis

## Abstract

**Objective:**

The relationship between frailty and breast cancer (BC) has been scarcely investigated. This study aims to explore the association between frailty and BC and to develop a predictive model for BC risk based on the frailty index (FI).

**Methods:**

This study utilized data from subjects with recorded frailty assessments and BC diagnosis collected by the National Health and Nutrition Examination Survey between 2003 and 2020. FI was assessed by 49 frailty indicators. Frailty was defined as FI > 0.20. First, logistic regression was used to analyze the link between frailty and BC. Subsequently, the dataset was divided into training and validation sets in a 7:3 ratio. Based on the training set, LASSO regression and 10-fold cross-validation were used to screen for the variables with the greatest predictive value. Then, the study built and evaluated eight machine learning (ML) models. Finally, SHapley Additive explanations was applied to interpret the best-performing model.

**Results:**

After data were excluded, a total of 4,473 patients were ultimately included. Logistic analysis revealed a positive correlation between FI and BC prevalence (*p* < 0.0001). Stratified analysis demonstrated that the correlation between the two variables was not influenced by other covariates. Five key feature variables were identified via LASSO regression and 10-fold cross-validation. Eight ML methods were employed to construct BC prediction models. After all the models were evaluated, the neural network demonstrated robust and reliable predictive performance and emerged as the optimal prediction model. Further the neural network model was elucidated via Shap, and the results demonstrated that the predictive variables ranked by importance from highest to lowest were age, FI, BMI, race, and education level.

**Conclusion:**

FI is positively correlated with the prevalence of BC. BC prediction model based on the attenuation index that this study developed and validated has high sensitivity, aids in early BC identification in frail individuals, and suggests that future studies refine its applicability.

## Introduction

Frailty is a state of decreased function across multiple organ systems, leading to reduced physiological reserve and resistance to stressors. It increases the risk of adverse health outcomes, including falls, disability, fractures, cardiovascular events, diabetes, cancer, and mortality (1–3). A report encompassing 62 countries reported that the prevalence of frailty among community-dwelling individuals ranges from 11% in those aged 50–59 years to 51% in those aged 90 years or older (2). As an emerging global health burden, frailty has significant implications for clinical practice and public health, garnering increasing international attention. The frailty index (FI), developed by Mitnitski et al. (4), Rockwood and Mitnitski (5), is a measure of the cumulative burden of health deficits. It is typically defined as the proportion of potential health deficits present in an individual. Numerous studies have shown that FI can predict all-cause mortality in older adults (6–8).

Globally, breast cancer (BC) is the most common malignant tumor among women. Numerous studies have indicated an association between frailty and BC (9–12). Wang et al. (11), reviewed literature on the prevalence of frailty in BC patients, reported a high prevalence of frailty among this population and that BC treatment may increase the risk of frailty. A retrospective cohort study by Mandelblatt et al. (13) revealed increased long-term all-cause mortality and BC-specific mortality among frail or prefrail patients. The adjusted hazard ratios for all-cause mortality (*n* = 209 deaths) were 1.7 (95% CI, 1.2–2.4) for prefrail women and 2.4 (95% CI, 1.5–4.0) for frail women compared with robust women, with an absolute mortality difference of 23.5%. The adjusted hazard ratio for BC deaths (*n* = 99) was 3.1 (95% CI, 1.6–5.8) for frail versus robust patients, with an absolute difference of 14%. Most of these studies have explored the prevalence of frailty and its correlation with mortality among patients diagnosed with BC. However, whether frailty is associated with BC prevalence remains largely unexplored. Furthermore, existing BC risk prediction models are predominantly based on clinical risk factors and imaging characteristics; they do not consider frailty status and are unable to specifically identify at-risk individuals within the frail population.

Machine learning (ML) holds significant promise for enhancing diagnostic processes by enabling the identification of hidden patterns and correlations within vast datasets through data-driven, automated, and precise decision-making (14). ML technology can be utilized to predict and diagnose BC by analyzing data sources such as medical imaging, patient demographics, and clinical records (15).

Therefore, the correlation between frailty and BC risk was analyzed via National Health and Nutrition Examination Survey (NHANES) data and created a predictive model based on FI to enhance the diagnosis and early screening of BC in the frail population.

## Methods

### Data source

The NHANES is a nationally represented survey program conducted by the National Center for Health Statistics (NCHS) under the Centers for Disease Control and Prevention (CDC). The NHANES database encompasses data across multiple domains. It primarily consists of the following components: health examination data, dietary assessment data, questionnaire data, and laboratory data. This database is designed to assess the health and nutritional status of adults and children in the United States, providing high-quality data that support the formulation of public health policies, health research, and epidemiological studies. Furthermore, the use of existing NHANES data for this analysis did not require additional approval from the Institutional Review Board. The NHANES data repository is accessible and publicly available on the NHANES website, facilitating broad use for research and policy development.

### Study population

**Figure 1:**
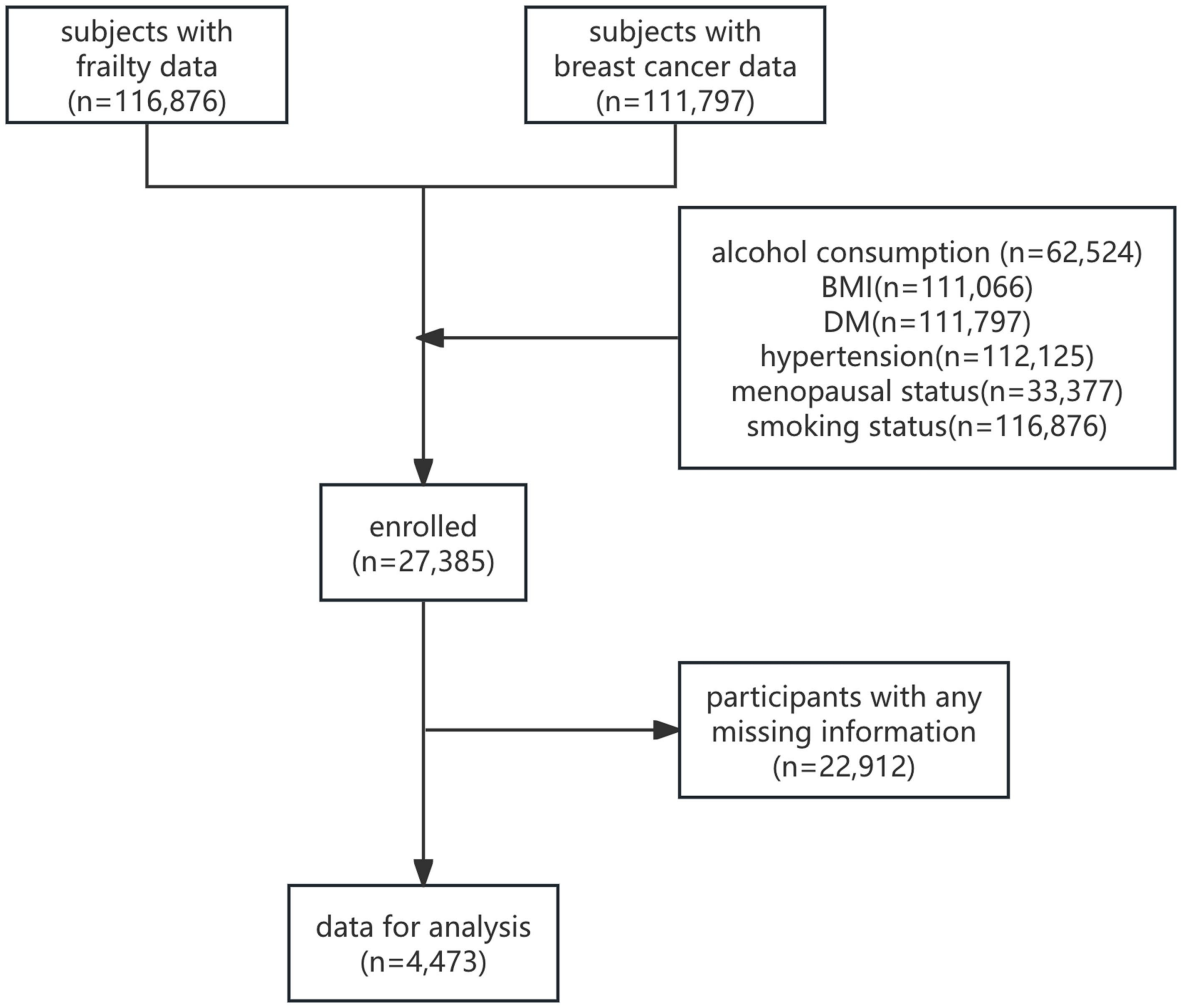
Flow chart of the selection of participants from NHANES 2003–2020.

Data for this study were obtained from NHANES surveys conducted between 2003 and 2020 (Figure 1). Initially, 116,876 subjects whose frailty data were available and 111,797 subjects whose BC data were available were included. The study incorporated multiple covariates, with the following data for each number of subjects: alcohol consumption (*n* = 62,524), BMI (*n* = 111,066), diabetes (*n* = 111,797), hypertension (*n* = 112,125), menopausal status (*n* = 33,377), and smoking (*n* = 116,876). After all covariates were merged and all subjects with any missing values were excluded (*n* = 22,912), a final cohort of 4,473 participants remained for analysis.

### Exposure variable

This study employed FI to assess an individual’s health status and degree of frailty. This study utilized 49 frailty indicators, which were selected on the basis of previous research and standardized protocols (16–18). The 49 indicators chosen represent a broad spectrum of health deficits, encompassing multiple domains, including physical (e.g., mobility limitation, fatigue), cognitive (e.g., memory problems), and functional (e.g., difficulties with activities of daily living) domains, as well as chronic diseases and laboratory abnormalities. These indicators were selected to ensure a comprehensive assessment of health deficits across different organ systems. If a specific deficit was present, the corresponding indicator was coded as 1; if it was absent, it was coded as 0. A continuous score ranging from 0 (no deficits) to 1 (deficits in all items), termed the FI, was calculated by dividing the total number of deficits present by the total number of indicators assessed. A higher score indicates a greater number of deficits and a greater risk of frailty. Furthermore, the FI was converted to a percentage by multiplying it by 100; then participants were categorized into nonfrail and frail groups using a cutoff point of 20%. This classification is derived from the standard construction procedure proposed by Searle et al. (18) and has been widely accepted internationally.

### Breast cancer data

In the “Medical Conditions” section of the NHANES questionnaires, information on the health conditions and medical histories of individuals was gathered, including the diagnosis of BC. The participants were asked the following questions: “Have you ever been told by a doctor or other health professional that you had cancer or a malignancy of any kind?”, followed by “What type of cancer was it?” Age at diagnosis was also collected with the following question: “How old were you when BC was first diagnosed?” The data mentioned above were reported only for women who were diagnosed with BC. Past verification showed that self-reported BC data had high specificity and medium sensitivity.

### Covariates

Demographic and clinical data obtained from the NHANES website were utilized as covariates. These variables included age; body mass index (BMI); fasting triglyceride level (mmol/L); fasting total cholesterol level (mmol/L); HDL cholesterol level (mmol/L); LDL cholesterol level (mmol/L); poverty-income ratio (PIR); menopausal status (no, yes); race/ethnicity (Eth: non-Hispanic white, Mexican American, non-Hispanic Black, Other Race); marital status (Mar: Married/Living with Partner, Widowed, Divorced, Separated, Never married); education level (EdU: Less than high school, high school diploma, More than high school); alcohol use status (Never, Former, Mild, Moderate, Heavy); smoking status (Never, Former, Current); hypertension (no, yes); and diabetes mellitus (DM: no, yes).

### Statistical analysis

This study exhibited an excessively high proportion of missing key covariates. However, since the imputation was based on a limited number of observations to infer most missing values, this approach may introduce more noise than useful information. Additionally, the missing data regarding alcohol consumption may not be random (e.g., individuals with high alcohol intake may avoid reporting), violating the random missingness assumption underlying imputation. Consequently, multiple imputation was not applied in this study.

Sampling weights were applied for the calculation of demographic descriptive statistics. For normally distributed continuous variables, the mean and standard deviation were calculated; for nonnormally distributed continuous variables, the median and interquartile range (IQR) were used. Categorical variables are presented as frequencies and percentages. To compare differences between groups, the independent samples *t* test was applied to normally distributed variables, whereas the Mann–Whitney *U* test was used for nonnormally distributed variables. Chi-square tests were employed for categorical variable comparisons. Logistic regression analysis was used to evaluate the association between FI and the prevalence of BC. To ensure the stability of results, the study performed stratified analysis according to BMI, smoking status, alcohol consumption status, hypertension, and diabetes. All descriptive statistics, logistic regression (19), and subgroup analyses were performed using the R survey package, incorporating NHANES sampling weights (adjusted for 2-year cycles), stratification variables (SDMVSTRA), and clustering variables (SDMVPSU).

### Feature selection

This study constructed a least absolute shrinkage and selection operator (LASSO) regression model to explore the relationships between all the variables and the prevalence of BC. LASSO regression controls model complexity by introducing a regularization parameter, lambda. Through L1 regularization of feature coefficients, LASSO can shrink the coefficients of certain features to zero, thereby performing feature selection. Then the LASSO model was optimized via 10-fold cross-validation and selected lambda_1se as the final regularization parameter for the definitive model.

### Model development

To develop BC prediction models and evaluate their predictive performance, the entire participant dataset was randomly split into a training set and a validation set at a 7:3 ratio. The selected variables were then used to construct models on the basis of the training set. A total of eight ML models were developed, including logistic regression, support vector machine (SVM), gradient boosting machine (GBM), neural network, eXtreme Gradient Boosting (XGBoost), adaptive boosting (AdaBoost), light gradient boosting machine (LightGBM), and categorical boosting (CatBoost).

### Model evaluation and interpretation

Given the relatively small proportion of BC cases in the dataset (approximately 4.3%), this study prioritized discrimination metrics (e.g., AUC) that are more robust against category imbalance as the primary evaluation criteria. The optimal statistical threshold for model performance was determined using the Jordon index, and a Decision Curve Analysis (DCA) was performed to visualize the clinical net benefit of the model. Furthermore, the accuracy, sensitivity, specificity, precision, and F1 score were calculated for each model to provide a comprehensive assessment of their performance. Confusion matrices were also plotted for each model to visually represent the classification results. Finally, all the models were validated via the validation set to evaluate their generalizability and predictive accuracy on an independent dataset. Model interpretation was performed via SHapley additive explanations (SHAP).

All analyses and modeling in this study were conducted via the statistical software R version 4.3.3 and RStudio with key packages including survey (v4.2–1), glmnet (v4.1–8), caret (v7.0–1), xgboost (v1.7.9.1), and shapviz (v0.9.3). All the statistical tests were two-sided, and a *p* value of less than 0.05 was considered statistically significant.

## Results

### Baseline characteristics

Table 1 presents the baseline characteristics of the study participants stratified by BC status. In this study cohort, approximately 4.3% (*n* = 194) of participants with frailty reported a history of BC. These participants were significantly older (69.54 ± 9.84 years vs. 61.90 ± 12.19 years, *p* < 0.0001) and had higher FI scores (22.22 ± 10.46 vs. 18.45 ± 10.38, *p* < 0.0001) than those without BC. Furthermore, a greater proportion of non-Hispanic white individuals (61.86%), never-smokers (58.25%), and individuals with diabetes mellitus (27.84%) were in the BC group.

**Table 1:** Survey-weighted, sociodemographic, and health status characteristics of participants by category of breast cancer in NHANES 2003–2020.

Characteristics	Total (*n* = 4,473)	Non-BC (*n* = 4,279)	BC (*n* = 194)	Statistic	*p* value
Frailty index, %	18.62 ± 10.41	18.45 ± 10.38	22.22 ± 10.46	−4.91	<0.0001
Age, years	62.23 ± 12.20	61.90 ± 12.19	69.54 ± 9.84	−10.46	<0.0001
BMI, kg/m^2^	30.09 ± 7.36	30.13 ± 7.38	29.27 ± 6.93	1.69	0.09
fast_triglyceride	1.37 ± 0.71	1.37 ± 0.71	1.46 ± 0.68	−1.79	0.08
fast_total_cholesterol	5.25 ± 1.10	5.24 ± 1.11	5.31 ± 1.03	−0.92	0.36
hdl_cholesterol	1.56 ± 0.43	1.55 ± 0.43	1.60 ± 0.45	−1.29	0.20
ldl_cholesterol	3.06 ± 0.98	3.06 ± 0.98	3.05 ± 0.96	0.18	0.85
PIR, %	2.11 (1.16, 4.00)	2.11 (1.15, 4.00)	2.08 (1.23, 4.22)	−0.92	0.36
Menopause, %				1.81	0.18
No	994(22.22)	959(22.41)	35(18.04)		
Yes	3,479(77.78)	3,320(77.59)	159(81.96)		
eth, %				25.36	<0.0001
White	1999 (44.69)	1879 (43.91)	120 (61.86)		
Mexican	622 (13.91)	603 (14.09)	19 (9.79)		
Black	985 (22.02)	951 (22.22)	34 (17.53)		
Other	867 (19.38)	846 (19.77)	21 (10.82)		
mar, %				3.77	0.58
Married	2,173 (48.58)	2071 (48.40)	102 (52.58)		
Widowed	1,051 (23.50)	1,004 (23.46)	47 (24.23)		
Divorced	612 (13.68)	586 (13.69)	26 (13.40)		
Separated	137 (3.06)	132 (3.08)	5 (2.58)		
Never married	372 (8.32)	362 (8.46)	10 (5.15)		
Living with partner	128 (2.86)	124 (2.90)	4 (2.06)		
edu, %				1.82	0.40
Under high school	1,146(25.62)	1,096(25.61)	50(25.77)		
High school	1,144(25.58)	1,102(25.75)	42(21.65)		
Above high school	2,183(48.80)	2081(48.63)	102(52.58)		
alcohol.user, %				3.13	0.54
Never	959 (21.44)	918 (21.45)	41 (21.13)		
Former	1,112 (24.86)	1,057 (24.70)	55 (28.35)		
Mild	1,335 (29.85)	1,274 (29.77)	61 (31.44)		
Moderate	656 (14.67)	633 (14.79)	23 (11.86)		
Heavy	411 (9.19)	397 (9.28)	14 (7.22)		
Smoking status, %				11.76	<0.01
Never	2,678 (59.87)	2,565 (59.94)	113 (58.25)		
Former	1,102 (24.64)	1,038 (24.26)	64 (32.99)		
Current	693 (15.49)	676 (15.80)	17 (8.76)		
Hypertension, %				2.97	0.08
No	1,680 (37.56)	1,619 (37.84)	61 (31.44)		
Yes	2,793 (62.44)	2,660 (62.16)	133 (68.56)		
DM, %				5.55	0.02
No	3,540 (79.14)	3,400 (79.46)	140 (72.16)		
DM	933 (20.86)	879 (20.54)	54 (27.84)		

### Associations between frailty and breast cancer

The results of the logistic regression analysis of the FI and the prevalence of BC are presented in Tables 2, 3. In Table 2, the FI is analyzed as a continuous variable. According to the crude model, compared with the low-FI group, the high-FI group presented a significantly greater prevalence of BC (OR = 1.03, 95% CI: 1.02–1.04). This finding was also confirmed in models adjusted for age and race (OR = 1.02, 95% CI: 1.01–1.04), as well as in models that accounted for all confounding factors (OR = 1.03, 95% CI: 1.02–1.05). In Table 3, FI is treated as a binary variable. Similarly, the high-FI group had a significantly greater prevalence of BC in the crude model (OR = 1.76, 95% CI: 1.32–2.35), as well as in the models adjusted for age and race (OR = 1.52, 95% CI: 1.13–2.05) and in the models adjusted for all confounders (OR = 1.71, 95% CI: 1.22–2.39). To ensure the stability of the link between frailty and the prevalence of BC, the study conducted subgroup analyses and interaction tests for BMI, smoking status, alcohol consumption status, hypertension, and diabetes. However, potential interactions between the above variables were not found (Tables 4, 5).

**Table 2:** Logistic regression analysis of FI (continuous variable) with the prevalence of breast cancer from NHANES 2003–2020 was performed.

Indicator	Crude		Model 1		Model 2	
	OR (95% CI)	*p* value	OR (95% CI)	*p* value	OR (95% CI)	*p* value
FI	1.03 (1.02,1.04)	<0.0001	1.02 (1.01,1.04)	<0.001	1.03 (1.02, 1.05)	<0.0001

Model 1 was based on age and ethnicity, and Model 2 was based on Model 1 plus BMI, poverty income ratio, marital status, education level, alcohol use, smoking status, hypertension, diabetes mellitus, menopause, fast_triglyceride_mmol.L, fast_total_cholesterol_mmol.L, and HDL_cholesterol_mmol. L, ldl_cholesterol_mmol.L.

**Table 3:** Logistic regression analysis of FI (binary variable) and the prevalence of breast cancer from NHANES 2003–2020.

Indicator	Crude		Model 1		Model 2	
	OR (95% CI)	*p* value	OR (95% CI)	*p* value	OR (95% CI)	*p* value
FI	1.76 (1.32,2.35)	<0.001	1.52 (1.13,2.05)	0.01	1.71 (1.22, 2.39)	0.002

**Table 4:** Stratified analyses of the associations between FI (continuous variable) and the prevalence of breast cancer in NHANES 2003–2020.

Subgroup	OR (95% CI)	*p*	*p* for interaction
Smoking status			0.65
Never	1.04 (1.02, 1.06)	<0.001	
Former	1.03 (1.00, 1.06)	0.04	
Current	1.03 (0.98, 1.08)	0.2	
Hypertension			0.13
No	1.05 (1.02, 1.08)	<0.001	
Yes	1.03 (1.01, 1.05)	0.003	
DM			0.87
No	1.03 (1.01, 1.05)	0.001	
DM	1.04 (1.01, 1.07)	0.004	

**Table 5:** Stratified analysis of the association between FI (binary variable) and breast cancer prevalence from NHANES 2003–2020.

Subgroup	OR (95% CI)	*p*	*p* for interaction
Smoking status			0.64
Never	1.86 (1.19, 2.90)	0.01	
Former	1.74 (0.95, 3.20)	0.07	
Current	1.07 (0.32, 3.52)	0.91	
Hypertension			0.08
No	2.70 (1.43, 5.05)	0.002	
Yes	1.42 (0.97, 2.11)	0.08	
DM			0.43
No	1.51 (1.02, 2.21)	0.04	
DM	2.62 (1.28, 5.86)	0.01	

### Predictor selection and model development

**Figure 2:**
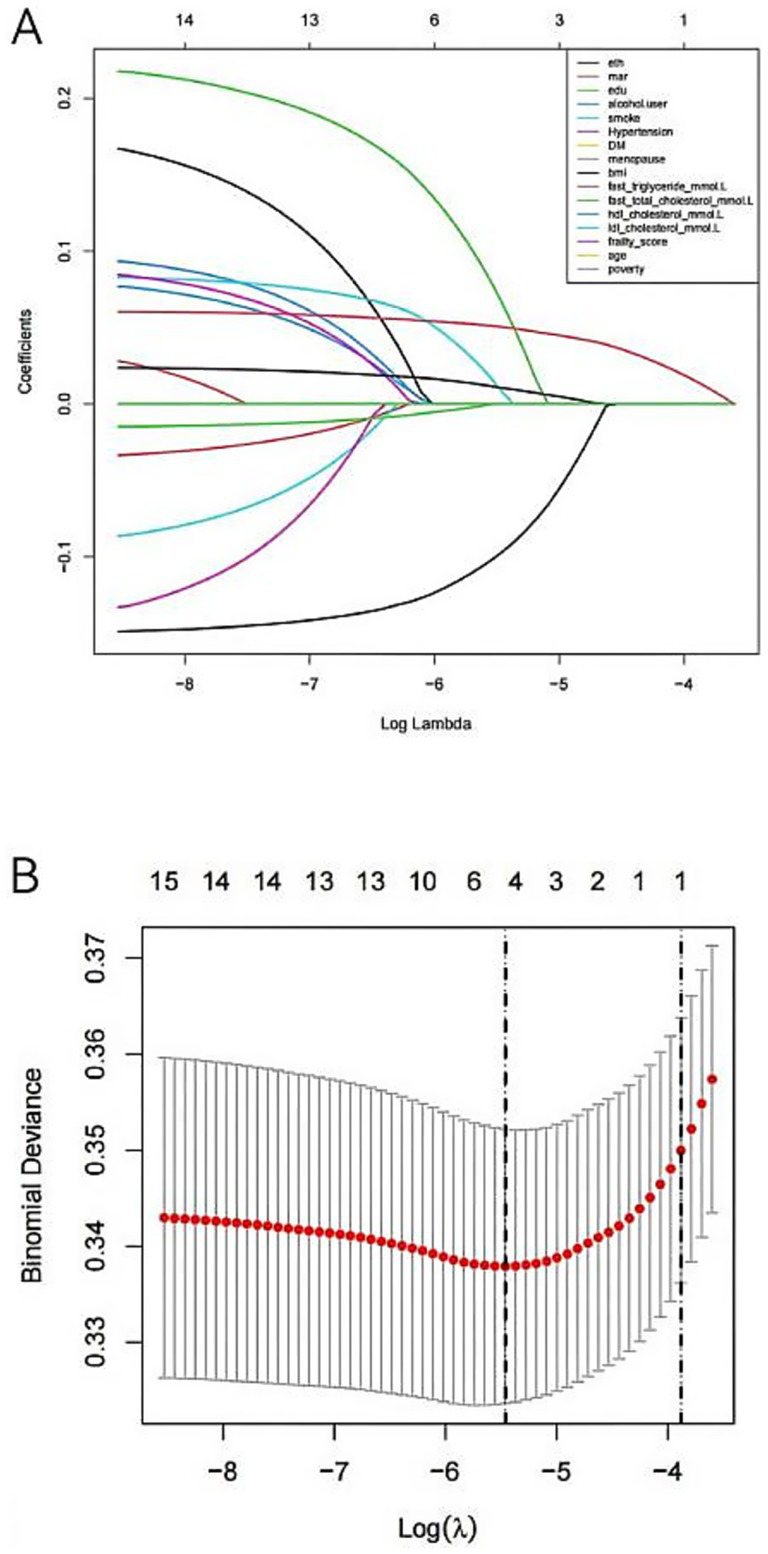
Path diagram for selecting variables for the LASSO regression model. **(A)** The horizontal axis is the log-transformed penalty parameter [log(*λ*)], and the vertical axis represents the corresponding regression coefficients. As the penalty parameter increases, the regression coefficients of each variable gradually approach zero. LASSO regression filters out variables with less predictive value by shrinking their coefficients to zero. **(B)** Results of the 10-fold cross-validation of the LASSO regression model. The horizontal axis shows the penalty parameter log(*λ*), and the vertical axis represents the binomial deviance. Red solid dots indicate the mean binomial deviance for each *λ* value, with vertical gray error bars representing the standard error. The numbers above correspond to the number of nonzero coefficients at each *λ*. This figure is used to evaluate model performance across different *λ* values and to select the optimal model and corresponding variables for construction.

To develop an optimal predictive model for BC based on FI, this study employed LASSO regression analysis to screen variables with significant predictive value for BC prevalence (Figure 2): race/ethnicity (Eth), education level (EdU), BMI, FI, and age. These predictors were subsequently used to construct a prediction model with eight ML algorithms.

### Model evaluation and interpretation

**Figure 3:**
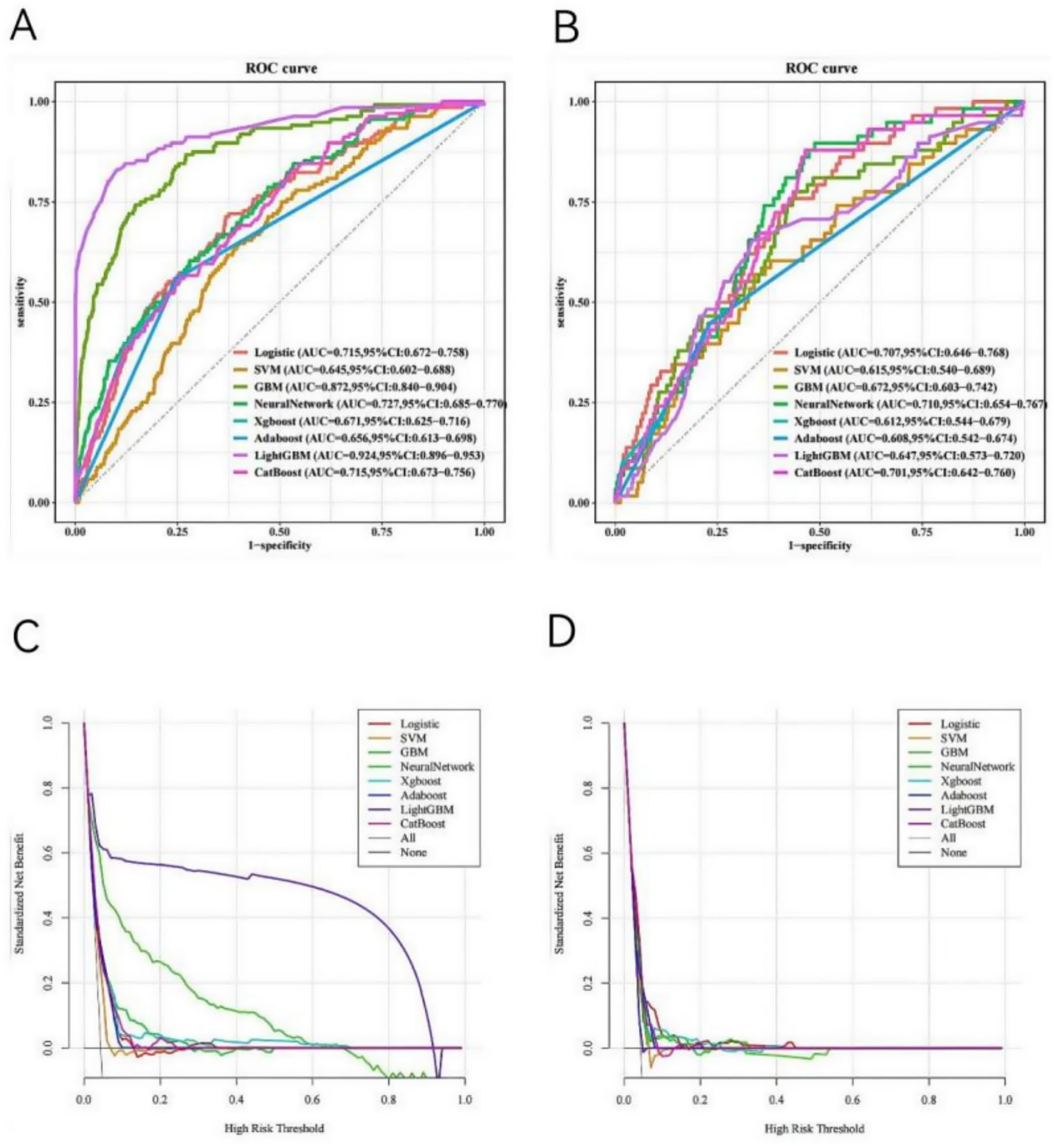
**(A)** Receiver operating characteristic (ROC) curves for eight ML models in the training set, along with their corresponding areas under the curve (AUCs). **(B)** ROC curves in the validation set. The horizontal axis represents 1-Specificity, and the vertical axis represents Sensitivity. The closer the ROC curve is to the upper left corner, the better the model’s classification performance. **(C)** Decision curves for eight forecasting models in the training set. **(D)** Decision curves for eight forecasting models in the validation set. The horizontal axis (X-axis) represents the threshold probability used in the model prediction—that is, individuals with a predicted probability greater than this cutoff are classified as positive. The vertical axis (Y-axis) represents net benefit, a metric that combines true positives and false positives predicted by the model in a weighted manner.

The low self-reported prevalence of BC in NHANES (about 4.3%) limits the number of positive events; based on 194 positive events and five predictive variables, with approximately 39 events per variable, the minimum criterion of ≥10 events/variable is met. Given the potential insufficiency of effective events when comparing eight models simultaneously, a conservative strategy in model selection was adopted, considering the consistency of performance between the training and validation sets, and performed hyperparameter tuning using 10-fold cross-validation to maximize sample utilization. The performance of these models is illustrated in Figure 3 via receiver operating characteristic (ROC) curves and decision curve analysis (DCA). Tables 6, 7 present the accuracy, sensitivity, specificity, precision, and F1 score for the training set and test set, respectively. Given the relatively low prevalence of BC (4.3%), the accuracy and F1 score remained low despite the application of category weights, which is expected.

**Table 6:** Model performance table for eight models on the training set; F1, harmonized average precision and recall rates.

Model	Threshold	Accuracy	Sensitivity	Specificity	Precision	F1
NA	NA	NA	NA	NA	NA	NA
Logistic	0.0401209198221286	0.629	0.721	0.624	0.08	0.144
SVM	0.0434270528539449	0.604	0.647	0.602	0.069	0.124
GBM	0.0392617617233933	0.736	0.868	0.73	0.127	0.222
NeuralNetwork	0.0442990925869749	0.715	0.603	0.72	0.089	0.155
Xgboost	0.495472460985184	0.744	0.559	0.753	0.093	0.159
AdaBoost	0.0862237438002229	0.744	0.559	0.753	0.093	0.159
LightGBM	0.0458371865064103	0.895	0.831	0.898	0.269	0.406
CatBoost	0.511463708684827	0.736	0.559	0.744	0.09	0.155

**Table 7:** Model performance table for eight models on the validation set; F1, harmonized average precision and recall rates.

Model	Threshold	Accuracy	Sensitivity	Specificity	Precision	F1
NA	NA	NA	NA	NA	NA	NA
Logistic	0.0348076568356461	0.596	0.741	0.589	0.075	0.137
SVM	0.0434301292804201	0.657	0.569	0.661	0.071	0.125
GBM	0.0257370381089579	0.569	0.776	0.56	0.074	0.135
NeuralNetwork	0.034689201332235	0.529	0.897	0.513	0.077	0.141
Xgboost	0.495472460985184	0.754	0.448	0.768	0.08	0.136
AdaBoost	0.0862237438002229	0.754	0.448	0.768	0.08	0.136
LightGBM	0.0110496935352384	0.664	0.655	0.665	0.081	0.144
CatBoost	0.510644369407255	0.55	0.879	0.535	0.079	0.145

After all the predictors from both the training and validation sets were integrated, the neural network model demonstrated the most robust and efficient predictive performance. When predicting BC in patients from the training set, the neural network model showed good discriminatory ability, with an AUROC of 0.727, an accuracy of 71.5%, a sensitivity of 60.3%, a specificity of 72%, and an F1 score of 15.5%. The model also exhibited strong performance in the validation set, with an AUROC of 0.710, an accuracy of 52.9%, a sensitivity of 89.7%, a specificity of 51.3%, and an F1 score of 14.1%. Compared with other ML models, LightGBM achieved the highest AUROC in the training set; however, the Neural Network attained the highest AUROC in the validation set. Upon comprehensive analysis, LightGBM was excluded because of a high likelihood of overfitting, which could lead to poor generalization to unseen data. Although logistic regression and CatBoost also demonstrated strong predictive performance, the neural network was deemed more stable across different datasets and was therefore selected as the optimal model.

**Figure 4:**
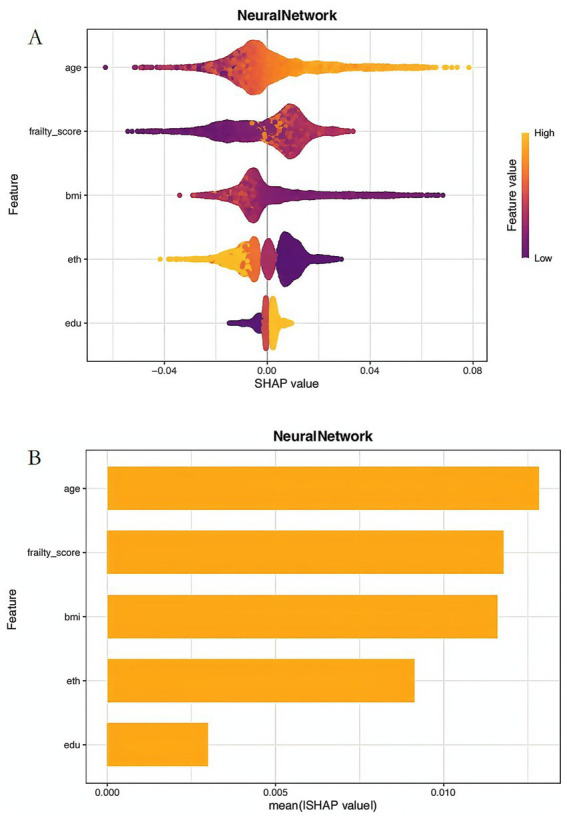
**(A)** SHAP Beehive diagrams for neural network models. The x-axis shows SHA*p* values, where higher values correspond to a greater positive influence of that feature on the prediction (i.e., increasing the likelihood of BC), whereas lower values correspond to a feature associated with a decreased likelihood. The color gradient from yellow to purple represents the magnitude of the feature value, with yellow indicating high values and purple indicating low values. Increased age and a higher frailty index were associated with an elevated risk of BC. **(B)** SHP bar chart showing the average absolute SHAp values of each feature in the neural network model for prediction.

Finally, SHAP was employed to interpret the model’s performance. Figure 4A illustrates the impact of age, FI, BMI, race, and education level on the output of the BC risk prediction model. Figure 4B displays the mean absolute SHAP values for each feature in the neural network model, which quantifies feature importance. This analysis revealed that age had the greatest impact among the evaluated features on the model’s predictions, followed by FI and BMI. Furthermore, FI was positively correlated with the prediction, indicating that higher FI values are correlated with higher BC prevalence rates.

## Discussion

By utilizing the large-scale NHANES database, this study established a positive association between FI and BC prevalence through correlation analysis, indicating that patients with a higher FI are more susceptible to developing BC. Furthermore, subgroup analyses demonstrated that this association is independent of key covariates, including BMI, smoking status, alcohol use, hypertension, and diabetes, identifying FI as a risk factor for BC beyond these factors. To further elucidate the predictive value of the FI for BC prevalence, LASSO regression was employed to identify five key predictors: frailty score, age, BMI, race, and education level. Subsequently, various ML methods were applied to construct eight distinct prediction models for BC using these predictors. A comparative evaluation revealed that the neural network model demonstrated strong and consistent predictive performance. It has emerged as the optimal predictive model, thereby providing a novel tool for the clinical prediction of BC.

Given the established association between frailty and mortality in BC patients, it is important to investigate whether frailty also serves as a predictor of BC prevalence. Chandler Coleman conducted a retrospective cohort study using the Surveillance, Epidemiology, and End Results-Medicare Health Outcomes Survey (SEER-MHOS) data resource for women aged 65 years and older who were diagnosed with first primary stage I-III BC between 1998 and 2013. Results indicated that older women exhibiting higher physical frailty and lower physical function faced the greatest risk of a stage III BC diagnosis. Consequently, he suggested that screening in older women with physical limitations should consider the increased risk of locally advanced BC (20). This study further confirms that the FI exhibits a significant correlation with BC, and dynamic monitoring of the frailty index should be implemented in debilitated populations in which regular BC screening is conducted. Frailty is closely related to immunosenescence, chronic inflammation, and cellular senescence (21). These factors can lead to impaired function of immune cells in the tumor microenvironment, impaired clearance of senescent cells, and abnormalities in apoptotic signaling pathways, thereby reducing the body’s ability to induce programmed cell death of tumor cells (22). These findings further support the core finding that FI is positively correlated with BC prevalence, and providing a theoretical basis for future interventions to enhance the efficacy of anti-tumor therapy by addressing frailty (23–25).

Generally, existing BC risk prediction models are primarily based on clinical risk factors, imaging characteristics, family history, and genetic mutations. Gail et al. (26) developed the Gail model, which initially incorporates five risk factors: age, age at first live birth, age at menarche, number of first-degree relatives with BC, and number of previous breast biopsies. This model can be used to estimate an individual’s absolute risk of developing BC over a 5-year period and over their lifetime. Women with a 5-year risk ≥1.67% are typically classified into a high-risk group. The US FDA recommends that high-risk women aged 35 years or older consider tamoxifen for risk reduction (27). The Breast Cancer Surveillance Consortium (BCSC) model is based on risk factors similar to the Gail model but additionally includes breast density as an independent risk factor, leading to improved predictive performance (28).

This study is the first to incorporate the frailty index into the BC risk prediction framework. By utilizing the NHANES population data system, the performance of eight ML models was compared, providing a new risk stratification tool for BC screening.

However, as an exploratory study, the current analysis still has several limitations: first, NHANES has a cross-sectional design, which cannot determine the temporal sequence between frailty assessment and BC diagnosis, posing a risk of reverse causality. Therefore, the model aims to assist in risk warning rather than infer causality. Second, the neural network model in the validation set has low specificity and a high false-positive rate, suggesting that it is more suitable as a preliminary screening signal to trigger further imaging examinations rather than serving as an independent diagnostic basis. Additionally, the proportion of positive events is relatively low, which meets the minimum standard of the rule of thumb for each predictive variable. However, the effective samples are still limited during multi-model comparison and hyperparameter tuning, which may affect model stability. Last, frailty may weaken the programmed death of anti-tumor cells through abnormalities in the immune senescence and apoptosis pathways. However, as a correlation analysis, this study fails to directly verify this biological mechanism.

Future external validation in a larger prospective cohort and further optimization of model performance by adjusting probability thresholds or incorporating multi-omics markers are needed.

## Conclusion

The analysis of NHANES data confirmed that FI is positively correlated with the prevalence of BC. LASSO regression was used to identify five predictive features and developed the first frailty index-based risk prediction model for BC. This model has high sensitivity and can serve as an auxiliary tool for preliminary BC risk assessment, but further improvement in precision or combination with other screening methods is needed before large-scale implementation.

## Data Availability

The datasets presented in this study can be found in online repositories. The names of the repository/repositories and accession number(s) can be found in the article/Supplementary material.
